# Findings from the Mighty Girls Efficacy Trial: Changes in Acceptance of Dating Violence

**DOI:** 10.3390/children11111331

**Published:** 2024-10-31

**Authors:** Michael L. Hecht, Anne E. Norris, Daniel Max Crowley, Jeff R. Temple, Hye Jeong Choi

**Affiliations:** 1 REAL Prevention LLC, 130 Pearl Brook Dr, Clifton, NJ 07013, USA; 2 School of Nursing & Health Studies, University of Miami, P.O. Box 248153, Coral Gables, FL 33124, USA; aen16@miami.edu; 3 College of Health and Human Development, Pennsylvania State University, 219 HHD Building, University Park, PA 16802, USA; dmc397@psu.edu; 4 Center for Violence Prevention, Betty and Rose Pfefferbaum Chair in Child Mass Trauma and Resilience, School of Behavioral Health Sciences, University of Texas Health Science Center, Houston, TX 77030, USA; jeffrey.r.temple@uth.tmc.edu; 5 College of Health Sciences, University of Missouri, 318 Clark Hall, Columbia, MO 65211, USA

**Keywords:** social emotional learning, sexual health, dating violence, Hispanic adolescents, prevention of risky sexual behaviors

## Abstract

Background/Objectives: Test efficacy of the social emotional learning (SEL)-based Mighty Girls program, a program culturally tailored for English-speaking Hispanic/Latino girls in seventh grade comprised of classroom sessions and a virtual reality computer game. We hypothesized that the curriculum would decrease risky sexual behaviors in a program that can be used as part of a comprehensive sex education curriculum or as a stand-alone program. Methods: A randomized group trial was conducted in which 22 low-income, predominately Hispanic schools within the Miami-Dade County Public School System were randomly assigned to intervention (consented *n* = 335) and control (consented *n* = 217) conditions. All study activities occurred after school. Primary outcome measures were resistance self-efficacy, acceptance of dating violence, sexual intentions, and sexual behavior. Assessments occurred at baseline, immediately post-intervention, 3-, 12-, and 24-months post-intervention. Changes in outcomes from baseline to 24 months were modeled using multi-level models to account for nesting of students within schools with full information maximum likelihood to account for missing data and baseline school attendance and enrollment in free and reduced lunch as covariates. Analyses are also controlled for multiple testing. Results: The program had a significant effect on reducing acceptance of dating violence at 24 months post-intervention (estimate = −0.083, *p* ≤ 0.05), but no effect on resistance self-efficacy, sexual intentions, or sexual behavior (*p* ≥ 0.58). Conclusion: Study findings demonstrate that a social emotional learning (SEL) curriculum can impact sexual behaviors such as susceptibility to dating violence. Low baseline levels for sexual intentions and behaviors as well as a high baseline of efficacy may have impacted findings for the other outcomes.

## 1. Introduction

While the overall teen birth rate in the United States has declined recently, rates among Hispanic adolescents remain high [1]. Indeed, national data indicate that proportionally more Hispanic teens give birth compared to their same-aged, non-Hispanic peers [1]. To effectively reduce teen pregnancies among this population, programs should be gender-specific and culturally reflective of Hispanic/Latino cultural values [2,3].

Early adolescence is a critically important developmental period characterized by rapid behavioral and biological growth, as well as the time when many health behaviors are acquired [4] and gender norms about behavior within romantic relationships are formed [5,6], Unsurprisingly, this is also the time when youth begin to experiment with risky behaviors. Thus, interventions delivered during this timeframe have the potential to make positive and enduring changes. Those that target multiple problematic behaviors—for example, unsafe sexual behavior and dating violence—may be especially promising [7]. Because attitudes supporting violence have been linked to both being a victim and perpetrator of dating violence [8,9], as well as risky sexual behavior [9,10] and teen pregnancy [11], pregnancy prevention programs should address attitudes about dating violence.

There are racial/ethnic disparities in these risks, with Black and Latina women more at risk for teen pregnancy [12] as well as for contracting HIV [13] and other sexually transmitted infections [14], which can lead to infertility and even death [12]. These experiences are then related to later problems and challenges, including failure to complete obtaining a high school diploma, incarceration, and unemployment [12]. Clearly, Latinx adolescents are at higher risk for negative sexual experiences. These risks appear heightened among recent Latina immigrants to Miami, where the study was conducted [15].

Another major developmental concern for early adolescents, regardless of culture/ethnic background, is peer acceptance [16], which makes tailoring interventions to address this concern critical to their effectiveness [17]. Indeed, peer influence has been shown to be a more powerful contributor to risky behavior than the buffering benefit of parental monitoring [18]. Unsurprisingly, research has found a consistent link between peer pressure, peer norms, and risky sexual behavior [19].

To guide intervention development, Mighty Girls is based on social emotional learning theory/SEL [20,21,22,23]. SEL argues that a focus on basic competences is needed to promote health development. The approach has been demonstrated to have wide-ranging effects across several domains by teaching basic skills (i.e., self-awareness, self-management, responsible decision making, relationship skills, and social awareness) across contexts (i.e., classrooms, schools, families and caregivers, and communities). The lessons teach the basic skills and are taught in an after-school context to facilitate the transfer of learning from schools to family and community settings. The implementation was highly interactive, with discussions, role plays, and games, along with some didactic content.

While pregnancy prevention interventions developed for Hispanic/Latino early adolescents typically include some of these components, they rarely address peer pressure directly or attitudes about dating violence. In contrast, the Mighty Girls program aims to build relationally competent communication skills for resisting peer pressure and media literacy skills for challenging messages supporting power imbalance and violence in male–female relationships [23]. The aim of this study is to develop an effective curriculum to curtail the participation of adolescents in risky sexual behaviors. Our primary hypotheses were that girls receiving Mighty Girls would report higher resistance self-efficacy, lower sexual intentions, less sexual behavior, and less positive attitudes about relationship violence at follow-up, relative to girls in the control condition. If successful, the project will provide an evidence-based intervention targeting risky sexual behavior among Latinas that has wide applicability due to its focus on sexual pressure resistance rather than more controversial aspects of sex education. Moreover, it will demonstrate the utility of narrative-based videogaming in health promotion and provide further support for the SEL approach.

## 2. Materials and Methods

### 2.1. Study Design

A group randomized trial design was implemented in 22 public schools within the Miami-Dade County Public School system (MDCPS) over a 2-year period. Assessments occurred at baseline, immediately after intervention in treatment schools, and at 3-, 12-, and 24-months post-intervention exposure. All study procedures were pre-approved by the University of Miami Institutional Review Board and the MDCPS Research Review Committee.

### 2.2. Randomization Process

Schools were randomized within enrollment year (“cohort”) and geographic area (upper, central, and lower Miami-Dade County). An odd number of schools within a cohort resulted in random assignment of the “extra” school to the intervention condition. The first year of enrollment (cohort 1) was comprised of five intervention and four control schools. The second year of enrollment (cohort 2) was comprised of eight intervention and five control schools. There were originally seven control schools, but two schools withdrew after randomization (immediately prior to starting recruitment while blind to study conditions). In total, the study consisted of 13 intervention and nine control schools.

#### 2.2.1. Recruitment and Study Participants

##### Schools

Schools with Hispanic enrollment greater than or equal to 60% and reduced/free lunch participation greater than or equal to 50% were eligible. Principals were free to agree or disagree with their school’s involvement in the study. Participating schools (C = 22) represent 58% of all MDCPS schools meeting inclusion criteria. PI, study staff, and schools were blind to school assignment until after study staff obtained parental consent.

##### Students

Student recruitment occurred during in-school assemblies of girls meeting study criteria, during which study staff distributed information packets containing parental consent forms. Staff also made these packets available in the school office. After receiving parental consent forms, study staff contacted parents of potential participants by phone to verify and review elements of informed consent, address any questions, and schedule participants. Finally, study staff obtained student informed assent.

The sample was comprised of 552 girls with consent and assent who also met participant inclusion criteria (at least one Hispanic or Brazilian parent or grandparent, enrolled in Grade 7, and English speaking) and did not meet any exclusion criteria (English for Speakers of Other Languages; developmental delay; severe hearing, vision, or speech impairment). Exclusion criteria were evaluated as part of obtaining parental consent over the phone using a checklist protocol.

**Figure 1:**
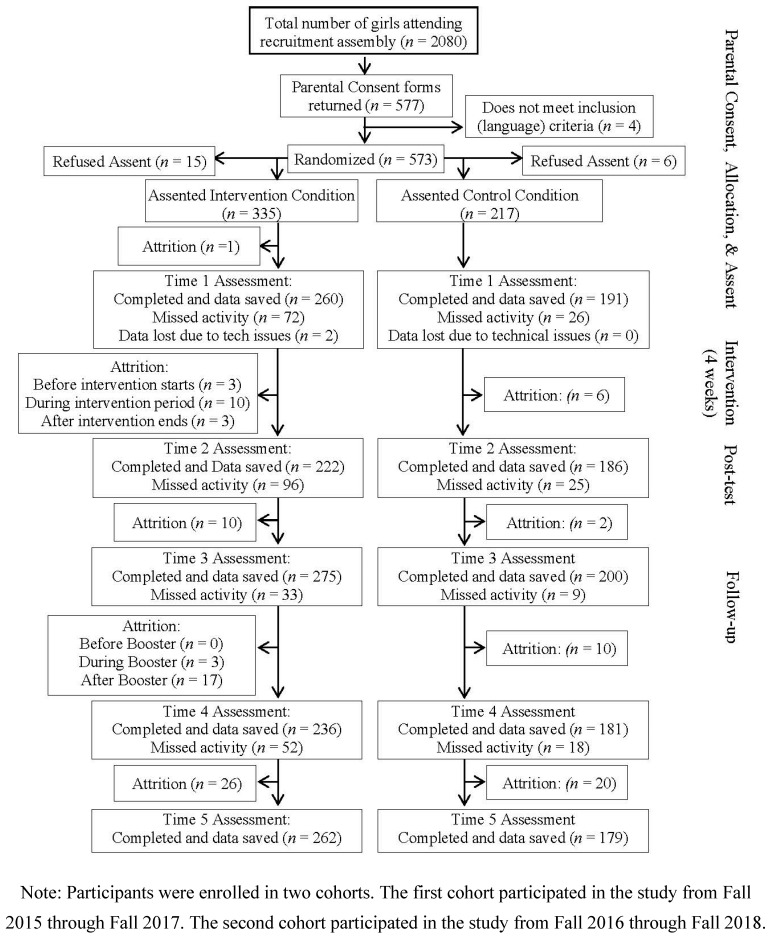
Consort diagram indicating flow of trial participants through study activities.

Enrollment rates, defined as the percentage of eligible girls with parental consent who agreed to participate in the study, were 23% and 29% for Cohorts 1 and 2, respectively. The percentage of girls whose parents consented but refused to provide assent was 4% and did not vary by study condition (*p* = 0.40). Figure 1 illustrates the progression of participants through study activities.

### 2.3. Data Collection

A survey containing study measures was completed electronically using a link provided by staff (all responses saved to a password-protected server using 128-bit SSL encryption). Survey software (LimeSurvey version 2.05) tailored item wording according to sexual orientation. Completion time varied (20–45 min) depending on reading ability and additional follow-up questions if vaginal intercourse or substance use was reported. Participants completed surveys after school, in a school classroom, using a Chromebook provided by study staff. For times 1 to 4, participants were supervised by staff. At time 5, students completed the survey at home as they were no longer at study schools (i.e., transitioned to high school). Participants unable to attend any of the initial four assessments were offered the opportunity to complete the survey at home. Staff assisting with at-home survey completion followed a procedure in which they first established the participant had privacy and sufficient time to complete the survey and provided a callback number in case there were difficulties. Once established, participants were provided a web link for the survey and a numerical code to access survey items (code used to link participant data across time points). Whether at school or at home, each assessment began with staff stressing the voluntary nature of participation. Participants received a $15 gift card and a community service hour for each survey they completed. This amount was deemed necessary to incentivize survey responses without being coercive. Dates of the study are 2015–2018. See Consort Diagram, Figure 1).

### 2.4. Study Conditions

#### 2.4.1. Intervention Condition

**Figure 2:**
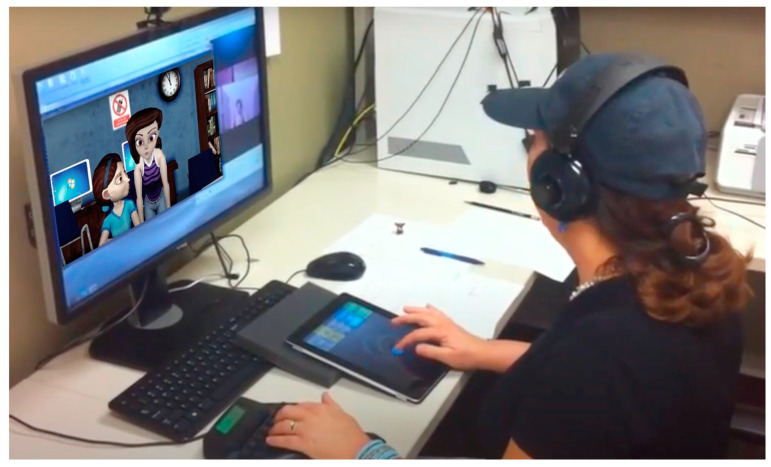
Interactor puppeting the avatars during Drama-Rama.

**Figure 3:**
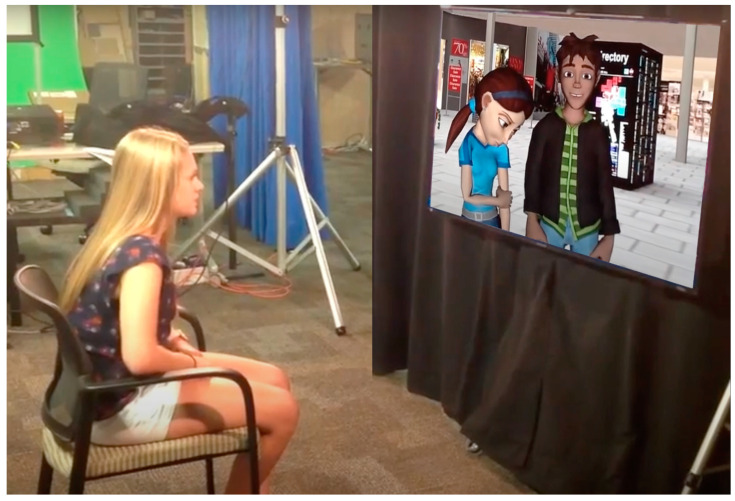
Girls playing Drama-Rama. ***Note:*** The young girl in Figure 3 is an actor, not a study participant.

Mighty Girls is a program for 12–13-year-old Hispanic/Latino girls consisting of six 45-min classroom sessions over a 2-week period followed by playing a 15-min virtual reality game (DRAMA-RAMA) twice during the two succeeding weeks. Sessions taught SEL skills to groups of 16–25 by female facilitators and co-facilitators selected for (a) experience teaching or working with groups of children in schools, summer camps, or afterschool programs, and (b) responses to questions about strategies for handling challenging classroom situations. All received an additional 28 h of training provided by the PI and project manager. The sessions were highly interactive and focused on risks and choices as well as communication and relationship skills. After completing the sessions, the girls participated in DRAMA-RAMA, a live virtual reality simulation of early adolescent social encounters involving peer pressure. It immerses the player in a situation derived from formative interviews in which they make choices requiring them to use the SEL skills taught during the classroom sessions. During the game, participants interacted with avatars “puppeted” by “interactors (female actors trained in use of technology and interactive performance) (see Figure 2). Game play occurred in small, private rooms, each equipped with a 42-inch TV monitor. A small Web camera was clipped on top of each monitor (see Figure 3). Games were played in English, one person at a time, for 15 min. DRAMA-RAMA used an object-oriented graphics rendering engine game engine. Audio interaction and video observation of the participant were delivered over the Internet via Skype. The Geppetto interface allowed the game to be played by talking (no keyboard or similar input required). The simulation was played twice as part of intervention, and twice 3 months later as intervention booster. See Table 1 for a more complete program description.

A total of 335 girls were enrolled in the intervention condition and 217 in the control condition. However, 8 of those in the intervention condition did not complete a survey at any study time point. All girls enrolled in the control condition completed at least one T1–T5 survey.

Mighty Girls was co-created by two of the authors with 2 of 6 classrooms and assistance, with sessions adapted from the evidence-based substance use prevention program, keepin’ it REAL (kiR) [23], that is listed as an SEL-certified program [24]. The acronym REAL stands for refuse, explain, avoid, leave, the names for four different resistance communication strategies that were identified in formative research and taught in kiR. Focused on building developing SEL skills, in particular self-awareness through goal setting, self-management through planning, responsible decision making, social awareness of peer pressure, and relationally competent peer resistance skills, kiR has proven effective, particularly among Hispanic/Latinos, as noted in the 2016 Surgeon General’s report on addiction [21].

While lesson content is based on SEL, the communication competence model (CCM) [19] and social cognitive theory (SCT) [25] guided the curriculum design of kiR and Mighty Girls. The CCM specifies how verbal and non-verbal communication skills can be used to communicate messages that leave the message recipient feeling positive about the message sender, even when the message is a refusal to go along with a request. CCM provides insights into how the SEL skills should be taught. It stresses that communication must be relationally competent—that is, effective for both parties. Teaching these communication skills is also congruent with Hispanic/Latino cultural values such as Personalismo and Simpatía, which emphasize protecting social harmony by having warm and conflict-free interactions [26,27]. SCT stresses that teaching SEL skills should be performed through models that increase a sense of efficacy. Combining the CCM and the SCT resulted in a behavior change program that builds resistance self-efficacy through modeling of specific communication skills.

Consequently, the program was also “adolescent-centered” because it addressed an outcome valued in early adolescence [28]. Program development was also informed by interactive focus groups [29] and consultation with various key informants and stakeholders, including Hispanic/Latino parents, Hispanic/Latino female high school students, a Planned Parenthood staff member, an Orlando public school’s public school administrator responsible for HIV prevention education, an active member of the National Organization of Women, and a Hispanic Seventh Day Adventist minister. Thus, the program was not only “adolescent-centered,” but also informed by formative work and responsive to stakeholder concerns.

#### 2.4.2. Control Condition

Participants received a website address for downloading Science Valley, a virtual reality game in which the goal is to solve “challenges” related to renewable energy, agriculture/sustainable living, and robotics. There were no classroom sessions for this condition, and game play occurred concurrently with intervention participants playing DRAMA-RAMA.

### 2.5. Instrument

The study survey included items assessing demographic and cultural characteristics, contamination, and potential outcomes (resistance self-efficacy, acceptance of dating violence, sexual intentions, sexual behaviors; Table 2). Resistance self-efficacy operationalizes our application of efficacy from Social Cognitive Theory, with the relevant belief being that they are capable of resisting sexual pressure. Acceptance of dating violence and sexual behaviors are the most salient outcomes of a sexual pressure resistance intervention, operationalizing the key behaviors we anticipated would result from the intervention. Sexual intentions were included as a predictor of these behaviors. All items were previously used with this age group in feasibility testing [19], work conducted with substance use prevention by study team members, or the CDC’s YRBS (Youth Risk Behavior Survey [30]). Feasibility testing items were pre-tested to ensure comprehension. See Table 2 for further detail about the measures.

### 2.6. Statistical Analyses

Descriptive analyses were conducted to characterize the sample and assess demographic variables. Baseline analyses comparing the control and intervention conditions were conducted. Multi-level outcome models evaluated the change in outcomes between the control and intervention conditions from baseline to T5. Analyses accounted for students nested within school and employed robust standard errors. Table 3 presents the baseline equivalences, and Table 4 presents the means across waves.

## 3. Results

### 3.1. Baseline Equivalence

No significant baseline differences were found between groups in study outcome variables (Table 3). Only three participants reported having had sexual intercourse at baseline, and all these participants were in the control condition. However, 101 intervention participants (30.2%) were missing on this item at baseline as compared to 37 (17%) in the control condition (*p* < 0.0001). See Table 3 for demographics.

### 3.2. Contamination

Control participant responses to close-ended questions indicated fairly minimal contamination occurring at T2 (*n* = 3), T4 (*n* = 5), and T5 (*n* = 9). Of the total number reporting hearing about the intervention (*n* = 17), only nine reported hearing about relationally competent resistance communication concepts, with three at T5 reporting hearing about all five listed intervention concepts. Higher reporting at T5 is consistent with participants from control and intervention schools mixing in high school. Including/excluding these participants in tests of study hypotheses had no effect on results of analyses.

### 3.3. Implementation Quality

#### 3.3.1. Dosage Received

Thirty-five percent (*n* = 116) of intervention participants attended all six classroom sessions, with 75% (*n* = 252) attending at least three. A majority (79%; *n* = 263) played DRAMA-RAMA twice, with half (51%; *n* = 171) receiving the booster (played four times). Participation was impacted by competing activities (e.g., doctor’s appointments, music lessons, school clubs or sports) and developmental concerns (e.g., friend(s) not wanting to attend, wanting to watch the boys’ basketball practice).

#### 3.3.2. Adherence

Facilitator classroom session adherence scores ranged from 3.6–5.7 (*mean* = 3.37, *SD* = 0.47) with a maximum of 6. Scores were comprised of points (0–2) for following lesson content, incorporating review of previously learned skills or content into session, and final session participant group performances and discussion in final session. Adding content not part of the curriculum resulted in a deduction of 1–2 points. DRAMA-RAMA adherence (percent of required game story plot elements and dialogue elements delivered) ranged from 66–100%, with 81% of sessions achieving 90–100% adherence.

#### 3.3.3. Quality of Program Delivery

Classroom session delivery quality scores ranged from 2 to 6 (*mean* = 5.60, *SD* = 0.34) with a maximum of 6. Scores were comprised of points (0–1) assigned for (a) making the session “Mighty Moment” (relationally competent communication kinesthetic learning activity developed by the project manager), (b) classroom climate having a “call and response” feel, (c) classroom management, (d) eagerness of class response, (e) friendly/fun feel, and (f) facilitator’s modeling of Mighty (relationally competent) communication. DRAMA-RAMA delivery quality was rated excellent on three different dimensions of quality using a 3-point scale (poor, average, excellent) for a large majority of game play sessions: (1) inter-actor performance energy (100%); (2) naturalness of conversation (98.5%); and (3) distinct and consistent game character characterization (95.7%).

### 3.4. Attrition

Overall, 20% of enrolled participants (*n* = 111) dropped or were lost to follow-up (i.e., phone disconnected, never returned phone calls, no longer enrolled at study school, hospitalized, or in foster care). Intervention condition participants (22%; *n* = 73) were not more likely to drop out of the study or be lost to follow-up than control condition participants (18%, *n* = 38); *p* > 0.19), and attrition did not alter comparability of intervention and control conditions at any time point (i.e., no differences with respect to receiving a reduced/free lunch, acculturation, being born in the United States, being of Cuban origin, generation, or puberty characteristics (*p* ≥ 0.65)). Attrition was also not greater in the intervention condition than in the control condition during the intervention period (*p* > 0.27). Reasons for attrition, including lost follow-up, did not differ significantly by study condition (*p* > 0.36) apart from providing no reason for dropping from the study (intervention: 7%, *n* = 24; control: 2%, *n* = 4; Fisher’s exact, *p* < 0.005).

### 3.5. Missing Data

There was no association between missing an assessment and receiving a reduced/free lunch, acculturation, being born in the United States, being of Cuban origin, immigrant generation, or menses onset (*p* > 0.47). However, intervention condition participants were more likely than those in the control condition to have missing data (*p* < 0.01). We experienced a significant differential rate of missingness amongst participants, with 44.31% of intervention participants compared to 30.88% of control participants missing data at the end of the study (estimate = 0.28, *p* < 0.001).

### 3.6. Tests of Study Hypotheses

The effect of the intervention on change in outcomes from baseline to 24 months was modeled using multi-level models to account for the nested structure of the data (students within schools), and baseline school attendance and enrollment in free and reduced lunch were included as baseline covariates. Proc Mixed in SAS 9.4 was employed to test models and assess intervention effects [35]. Full information maximum likelihood procedures were employed to account for missing data [36].

**Figure 4:**
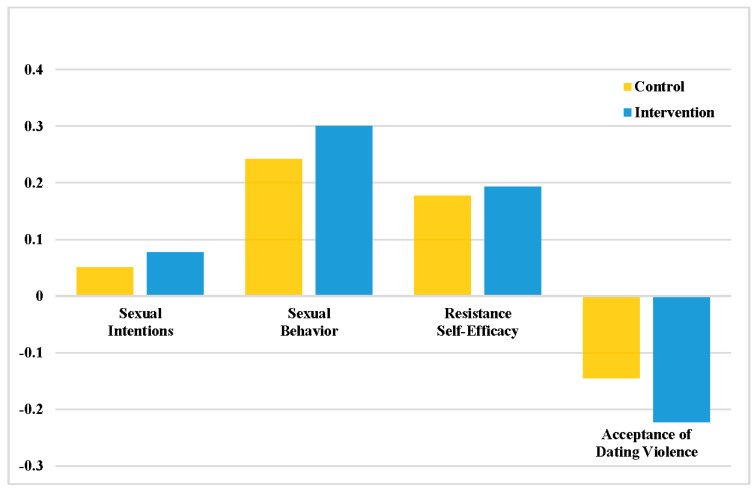
Change scores (difference between baseline and T5) in outcomes.

No significant baseline differences were found between groups on sexual intentions (*estimate* = 0.041, *p* = 0.58), sexual behavior (*estimate* = −0.01, *p* = 0.99) or self-efficacy (*estimate* = 0.014, *p* = 0.80). A significant effect was found for acceptance of dating violence, with intervention condition participants less likely to support dating violence than those in the control condition 24-months post-intervention (*estimate* = −0.083, *p* ≤ 0.05). Figure 4 displays the amount of change in study outcome variables observed in each study condition over a 24-month period. The Y-axis is the difference of means of the study variables between time points. Additional statistical information is provided in Appendix A.

## 4. Discussion

The aim of this study was to develop an effective program for reducing risky sexual behaviors among adolescents. Contrary to expectations and results from a previous feasibility trial [19], we were unable to detect program effects on self-efficacy and sexual behavior. Thus, the prediction about efficacy from social cognitive theory is not confirmed. We suspect the analysis was underpowered due to ceiling effects in our self-efficacy data resulting from the observed skewing of responses (i.e., the majority of responses were strongly agree or agree) and low baseline rates of sexual intercourse, which may reflect under-reporting of sexual behavior. The rates of sexual behavior we obtained were lower than those reported by Hispanic female participants in Miami-Dade County YRBS middle and high school surveys [30] (Centers for Disease Control and Prevention [CDC], 2020), which may only partly be explained by when we collected data (fall semester for time 4 and time 5) vs. YRBS (spring semester). It is also possible that the voluntary nature of the sample resulted in a study population that was less risky than the general school population of Hispanic girls enrolled in Miami-Dade County public schools.

We did find evidence for a delayed effect of the Mighty Girls program on attitudes about relationship violence, suggesting that the program may influence teen dating violence as participants grow into later adolescence and emerging adulthood. This effect emerged at the final time point (fall semester of 9th grade), possibly reflecting the increased dating pressure and expectations often witnessed with the transition to high school. Interestingly, only one intervention session addressed violence in relationships, and this was in the context of images in the media about male–female relationships. However, using relationally competent communication skills to assert your opinion, refuse a request, and resist peer pressure was stressed in every session. Thus, this finding argues for the potential power of teaching relationally competent communication skills in combination with media literacy skills regarding the portrayal of male–female relationships in advertising. It also supports the application of the Communication Competence Model to the intervention by demonstrating a protective effect through the communication and relationship skills imparted during the lessons.

Our study findings generate a critical question for future research on the impact of SEL interventions that promote relationally competent communication on conflict resolution, involvement in violent relationships, and safer sex negotiation. Building personal (decision making), communication (resistance strategies), and relationship (conflict reduction) skills should foster healthy relationships by promoting knowledge and efficacy. Theoretically, increasing these SEL competencies should manifest themselves in healthier lives. However, interventions do not exist in isolation, and focusing on changing individual behavior is limited by the outer layers of the social ecology. It is incredibly difficult to overcome the unhealthy environments and models (i.e., social media content) that often surround our youth (including current participants). Thus, we believe the next logical step is a larger study that (1) allows subgroup analyses to determine if Mighty Girls and similar programs work better for some groups than others and (2) considers additional components that address multiple levels of social influence in the SEL model (e.g., relationship, family, community). Further, effective interventions delivered during early adolescence may be particularly beneficial to shape how people relate prior to the development of unhealthy behavioral patterns. Such interventions can have a growing positive impact across the life span because skills and ideas learned in early adolescence may become enduring patterns that influence future behavior [12]. However, demonstrating such effects requires a longer framework for longitudinal analyses that follow youth as they age into adulthood.

A second critical question is the viability of virtual reality games such as DRAMA-RAMA. Not only is the technology expensive, costs and implementation complexity are exacerbated by the “human element” in the form of interactants introduced to maximize realism and interactivity. VR headsets are now available to replace the TV, but space is still required for privacy, and the authors are not convinced that the affordances of AI will adequately provide the nuance needed during game play that was gained from the interactants puppeting the avatars. It is possible that a simpler technology may suffice. For example, a narrative writing app would allow participants to construct stories about pressure resistance and SEL skill enactment that not only reinforces skill development but teaches narrative writing.

### Limitations

There are several limitations of this study. First, all outcome data are self-reported, which likely resulted in the underreporting of sexual behavior through issues like social desirability and the capacity for self-perception. We attempted to mitigate this concern by employing computer-delivered assessments, which have been shown to encourage greater self-disclosure by adolescents relative to paper/pencil assessments [37]. In addition, intervention activities did not describe, call out, or focus on specific sexual behaviors except for “kissing a boy you did not know very well” in the first DRAMA-RAMA game. Finally, staff were not involved with collecting survey data at any school in which they delivered intervention sessions. Nevertheless, the observations of staff involved with assessments indicated dislike or discomfort with the sexual behavior items (e.g., participants complained they were inappropriate).

Second, missing data occurred, with significant differential missingness between study conditions (i.e., in the intervention condition, approximately 25% of girls did not attend half the sessions), which likely contributed to difficulty detecting intervention effects. It is also possible that the various causes of missing data (e.g., technical issues, missing assessments, missing/refusing to answer a survey item, attrition) may have unrecognized effects on study outcomes. However, we employed recommended methods for handling missing data [38], which enabled us to adhere to intention to treat (TIT) principles by retaining all participants in the analysis within the groups to which they were originally randomized, irrespective of their participation in intervention activities, completion of data collection sessions, or attrition [39]. Adhering to ITT principles maintains comparability of study conditions obtained through randomization and eliminates bias [40] and produces conservative estimates of treatment effects [41]. This argues for the trustworthiness of study results. It is, perhaps, endemic to designs implemented outside of school that youth data will include high degrees of missingness. The “intent-to-treat” model, while standard, does reduce power. Designs where data are collected under more controlled circumstances (e.g., during school) would decrease this limitation.

Related to missing data was the apparent hesitancy of many parents to provide signed consent. Topics related to sex are controversial and have become increasingly politicized in the United States and particularly in the state of Florida. It is likely the most religious and/or conservative parents were overrepresented in the non-consented group, providing a potential bias in the sample.

Third, the effect size for even the significant finding for attitudes about relationship violence was small. That said, a finding for females this young puts them on a path to healthier sexual relations, so even a small effect can have practical significance. The low baseline levels of risk behaviors in this study limited effects and demonstrated the potential for even greater impact on groups with higher levels of risk behaviors.

Fourth, the construct validity of the measures was not tested. Although previously validated measures were used with low-income Hispanic girls in Florida and other parts of the US, this may still be an issue regarding the accuracy of the measurement and the findings.

Fifth, the goal was to assess the change between baseline and final measurement. As a result, longitudinal analyses are not reported. Earlier posttests occurred during middle school, a time of limited sexual exploration. Since long-term effects are the strongest indicator of program outcomes, we chose to compare the T5 assessment, approximately 2 years after the intervention and during high school, to baseline.

## 5. Conclusions

This study addressed the question of how we can improve the sexual health of adolescents through effective prevention interventions. The Mighty Teens program was developed based on social emotional learning theory and tested in a group randomized trial to examine its efficacy. While all the expected results were not realized, the Mighty Girls intervention demonstrated effects on sexual behavior in a previous randomized trial in Orlando [19] and effects on attitudes about relational violence in the present study. Additionally, Mighty Girls has the advantage of being a sexual pressure resistance intervention that focuses on SEL skill building rather than reproductive health content. This allows the program to surmount the political and ideological issues surrounding sex education that create barriers to program implementation [42]. While not meant as a comprehensive sex education curriculum, study findings argue for Mighty Girls having demonstrated the potential to impact issues of sexual health, including teen pregnancy and STIs, through its effect on attitudes about teen dating violence.

## Figures and Tables

**Table 1:** Mighty Girls program components.

Lesson	Content
1. Choices and Results	Personal goals, everyday choices, and consequences related to goals
2. What’s Risky	Risky behaviors, consequences of risky behaviors, personal risk
3. Avoid Skill ^a^	Define the avoid skill, 3 methods to avoid pressure, and the Mighty Girl way of avoiding
4. Refuse Skill ^a^	Aggressive, passive, and assertive communication, matching verbal and nonverbal communication, and the Mighty Girl way of refusing
5. Media Influences	Define media, understanding media influence, positive and negative media messages about girls, and the difference between media teens and “real” teens.
6. Wrap-up and Review	Results-based choices, practice Mighty Girl skills
DRAMA-RAMA ^b^	Live videogame simulation of peer pressure with participants deciding whether or not to engage in risky behaviors, practice of Mighty Girls skills

^a^ Adapted from keepin’ it REAL [23]; ^b^ DRAMA-RAMA was played twice as part of the intervention, and twice 3 months later as an intervention booster.

**Table 2:** Outcome measures and respective Cronbach’s alpha statistic.

Construct	Number of Items	Sample Item	Response Options	Source	T1-T5 Cronbach’s Alpha Values
Resistance Self-Efficacy	16	Stop someone who is pressuring you to do sexual things without making them angry.	5-point scale with labels for mid- and endpoints only (0 = Not at all sure I can do this; 2 = Moderately; Sure, I can do this; 4 = Completely; Sure I can do this	Adapted from DiIorio et al. [19,31]	0.92–0.94
Sexual Intentions	4	I would have sex now if a girl ^a^ I cared about pressured me to have sex.	4-point scale (0 = No, definitely not; 3 = Yes, definitely yes)	Kirby et al. [32]	0.87–0.92
Sexual Behavior	11	Had a boy ^b^ touch you below the waist, underneath your clothing.	5-point scale (0 = Never; to 4 = 10 times or more)	[33]	0.85–0.93
Acceptance of Dating Violence	6	A girl who makes her boyfriend jealous on purpose deserves to be hit ^c^	4-point scale (1 = strongly disagree; 4 = strongly agree)	Foshee et al. (1998) [34]	0.80–0.84

^a^ Same-sex orientation wording. Item tailored for opposite sex orientation using “boy.” Item tailored for bisexual orientation, and unsure or refused to answer responses using “boy or girl.”. ^b^ Opposite sex orientation wording. ^c^ This item is not tailored for sexual orientation.

**Table 3:** Baseline demographics for intervention and control condition participants ^1^.

Variable	Intervention (*n* = 325)	Control (*n* = 217)
Age in years		
Mean (SD ^a^) Median (range)	12.31 yrs. (0.68) 12 (11–15)	12.36 yrs. (0.78) 12 (11–15)
Acculturation Score		
Mean (SD) Median (range)	3.73 (0.72) 3.75 (1.5–5.3)	3.73 (0.64) 3.75 (1.5–5)
Born in US	71%	70%
Generation ^b^		
Mean (SD) Median (range)	2.03 (0.88) 2 (1–4)	1.9 (0.78) 2 (1–4)
Country of Origin ^c^		
Cuba	40%	43%
Mexico	6%	<1%
Dominican Republic	3%	3%
Puerto Rico	2%	<1%
Central America	12%	15%
South America	10%	9%
Multiple Countries	27%	29%
Reduced/Free Lunch	86%	82%
Started Menses	62%	58%

Note. ^1^ Group means compared using the Student’s t-test. Group proportions (i.e., percentages) were compared using the chi-square test. No significant group differences were observed in any demographic characteristics (*p* ≥ 0.10). ^a^ SD = standard deviation. ^b^ Generation is defined as follows: 1 = Participant not born in the US (first generation). 2 = Participant is first person in family born in the US (second generation). 3 = Participant and at least one parent born in the US (third generation). 4 = Participant and at least one parent and at least one grandparent born in the US (fourth generation). ^c^ Chi-square analysis compared the proportion of Cuban Americans in each group against all other countries of origin combined.

**Table 4:** Means across waves.

Variable	Wave	Control		Intervention	
		Mean	Std Dev	Mean	Std Dev
Acceptance of Dating Violence	1	1.27	0.39	1.33	0.39
	2	1.22	0.35	1.25	0.32
	3	1.24	0.41	1.20	0.32
	4	1.17	0.28	1.17	0.25
	5	1.14	0.28	1.13	0.23
Resistance Self-Efficacy	1	4.48	0.79	4.48	0.68
	2	4.56	0.70	4.57	0.69
	3	4.58	0.71	4.60	0.62
	4	4.63	0.55	4.59	0.56
	5	4.64	0.58	4.67	0.48
Sexual Intentions	1	0.10	0.34	0.09	0.30
	2	0.09	0.26	0.13	0.44
	3	0.10	0.33	0.10	0.34
	4	0.08	0.28	0.14	0.37
	5	0.15	0.39	0.17	0.37
Sexual Behavior	1	0.13	0.36	0.22	0.47
	2	0.15	0.34	0.21	0.44
	3	0.22	0.53	0.24	0.50
	4	0.26	0.49	0.44	0.78
	5	0.45	0.73	0.60	0.82

## Data Availability

The data presented in this study are available on request from the second author. The data are not publicly available due to restrictions privacy.

## Appendix A

**Table A1:** Baseline and 24-month follow-up means for self-efficacy, sexual intentions, sexual behavior, and acceptance of dating violence by study condition.

Variable	Intervention Condition		Control Condition	
	T1 Oct-Dec 2015	T5	T1	T5
Resistance Self-Efficacy	4.48 (0.68) Range: 1–5 (*n* = 251)	4.47 (0.49) Range: 2–5 (*n* = 261)	4.48 (0.73) Range: 1–5 (*n* = 187)	4.64 (0.528) Range: 1–5 (*n* = 177)
Sexual Intentions	0.09 (0.30) Range: 0–3 (*n* = 244)	0.17 (0.37) Range: 0–1.80 (*n* = 260)	0.10 (0.34) Range: 0–3 (*n* = 185)	0.15 (0.39) Range: 0–3 (*n* = 179)
Sexual Behavior	0.22 (0.47) Range: 0–2.58 (*n* = 236)	0.60 (0.82) Range: 0–4 (*n* = 259)	0.13 (0.36) Range: 0–3.83 (*n* = 181)	0.45 (0.73) Range: 0–4 (*n* = 177)
Acceptance of Dating Violence	1.33 (0.39) Range: 1–3 (*n* = 260)	1.13 (0.23) Range: 1–2.56 (*n* = 261)	1.27 (0.39) Range: 1–4 (*n* = 190)	1.14 (0.28) Range: 1–2.72 (*n* = 179)

**Table A2:** Resistance self-efficacy model.

**a. Solution for Fixed Effects:**					
**Effect**	**Estimate**	**Standard** **Error**	***df***	***t* Value**	**Pr > |*t*|**
Intercept	−0.7871	1.3310	19	−0.59	0.5612
Study Condition	−0.00069	0.08768	314	−0.01	0.9937
Student Qualifies for Free/Reduced Price Lunch	0.03232	0.06698	314	0.48	0.6298
School’s Average Daily Attendance	0.9443	1.3965	314	0.68	0.4994
**b. Type 3 Tests of Fixed Effects**					
**Effect**			***df***	***F* Value**	**Pr > *F***
Study Condition			1, 314	0.00	0.9937
Student Qualifies for Free/Reduced Price Lunch			1, 314	0.23	0.6298
School’s Average Daily Attendance			1, 314	0.46	0.4994

**Table A3:** Sexual intentions model.

**a. Solution for Fixed Effects**					
**Effect**	**Estimate**	**Standard** **Error**	***df***	***t* Value**	**Pr > |*t*|**
Intercept	−0.01458	0.7507	19	−0.02	0.9847
Study Condition	0.01232	0.05155	315	0.24	0.8113
Student Qualifies for Free/Reduced Price Lunch	−0.02878	0.03467	315	−0.83	0.4070
School’s Average Daily Attendance	0.1537	0.7897	315	0.19	0.8458
**b. Type 3 Tests of Fixed Effects**					
**Effect**			***df***	***F* Value**	**Pr > *F***
Study Condition			1, 315	0.06	0.8113
Student Qualifies for Free/Reduced Price Lunch			1, 315	0.69	0.4070
School’s Average Daily Attendance			1, 315	0.04	0.8458

**Table A4:** Sexual behavior model.

**a. Solution for Fixed Effects**					
**Effect**	**Estimate**	**Standard** **Error**	***df***	***t* Value**	**Pr > |*t*|**
Intercept	0.02674	0.9952	19	0.03	0.9788
Study Condition	0.04129	0.06870	297	0.60	0.5483
Student Qualifies for Free/Reduced Price Lunch	0.01131	0.04688	297	0.24	0.8096
School’s Average Daily Attendance	0.2036	1.0470	297	0.19	0.8459
**b. Type 3 Tests of Fixed Effects**					
**Effect**			***df***	***F* Value**	**Pr > *F***
Study Condition			1, 297	0.36	0.5483
Student Qualifies for Free/Reduced Price Lunch			1, 297	0.06	0.8096
School’s Average Daily Attendance			1, 297	0.04	0.8459

**Table A5:** Acceptance of dating violence model.

**a. Solution for Fixed Effects**					
**Effect**	**Estimate**	**Standard** **Error**	***df***	***t* Value**	**Pr > |*t*|**
Intercept	−0.5550	0.6356	19	−0.87	0.3934
Study Condition	−0.08281	0.04160	324	−1.99	0.0473
Student Qualifies for Free/Reduced Price Lunch	0.04165	0.03150	324	1.32	0.1871
School’s Average Daily Attendance	0.3125	0.6672	324	0.47	0.6399
**b. Type 3 Tests of Fixed Effects**					
**Effect**			***df***	***F* Value**	**Pr > *F***
Study Condition			1, 324	3.96	0.0473
Student Qualifies for Free/Reduced Price Lunch			1, 324	1.75	0.1871
School’s Average Daily Attendance			1, 324	0.22	0.6399
